# Supplementary material for: Involvement of the SnRK1 subunit KIN10 in sucrose-induced hypocotyl elongation
Source: Plant Signal Behav. 2018 May 30;13(6):e1457913. doi: 10.1080/15592324.2018.1457913 (PMC6110359; doi:10.1080/15592324.2018.1457913)
Supplement: Supplemental Material [file kpsb-13-06-1457913-s001.zip › Simon et al Revised.docx]

**Involvement of the SnRK1 subunit KIN10 in sucrose-induced hypocotyl elongation**

Noriane M. L. Simon^a^, Ellie Sawkins^a^ and Antony N. Dodd^a*^

^a^School of Biological Sciences, University of Bristol, Life Sciences Building, 24 Tyndall Avenue, Bristol BS8 1TQ, U.K.

* Corresponding author; antony.dodd@bristol.ac.uk

Word count: 857

**Keywords**: Arabidopsis, signal transduction, metabolism, development.

**Abstract**

A mechanism participating in energy sensing and signalling in plants involves the regulation of sucrose non-fermenting1 (Snf1)-related protein kinase 1 (SnRK1) activity in response to sugar availability. SnRK1 is thought to regulate the activity of both metabolic enzymes and transcription factors in response to changes in energy availability, with trehalose-6-phospate functioning as a signalling sugar that suppresses SnRK1 activity under sugar-replete conditions. Sucrose supplementation increases the elongation of hypocotyls of developing Arabidopsis seedlings, and this response to sucrose involves both the SnRK1 subunit KIN10 and also TREHALOSE-6-PHOSPHATE SYNTHASE1 (TPS1). Here, we measured sucrose-induced hypocotyl elongation in two insertional mutants of KIN10 (*akin10* and *akin10*-2). Under short photoperiods, sucrose supplementation caused great proportional hypocotyl elongation in these KIN10 mutants compared with the wild type, and these mutants had shorter hypocotyls than the wild type in the absence of sucrose supplementation. One interpretation is that SnRK1 activity might suppress hypocotyl elongation in the presence of sucrose, because KIN10 overexpression inhibits sucrose-induced hypocotyl elongation and *akin10* mutants enhance sucrose-induced hypocotyl elongation.

**Main text**

We reported recently the involvement of a sugar-signalling mechanism in a pathway that causes hypocotyl elongation in response to sucrose^1^. Hypocotyl elongation in *Arabidopsis thaliana* (Arabidopsis) seedlings is caused by cell expansion within the elongating hypocotyl and represents an informative experimental model to study signalling processes that regulate development. In Arabidopsis, hypocotyl length is increased by supplementation of the growth media with sucrose^2-9^. We identified that the sugar- and energy-sensing kinase sucrose non-fermenting1 (Snf1)-related protein kinase 1 (SnRK1) regulates sucrose-induced hypocotyl elongation^1^. Under short photoperiods, hypocotyls did not elongate in response to exogenous sucrose in seedlings overexpressing the catalytic alpha subunit of SnRK1, termed SNF1-RELATED PROTEIN KINASE1.1 (KIN10/AKIN10/SnRK1.1)^1^. We also found that TREHALOSE-6-PHOSPHATE SYNTHASE1 (TPS1) is required for sucrose-induced hypocotyl elongation under short photoperiods^1^. TPS1 synthesizes the sugar trehalose-6-phosphate (Tre6P), which is a potent inhibitor of SnRK1 activity^10^. Tre6P is thought to function as a signalling sugar that provides information about cellular energy availability^10, 11^.

Hypocotyl elongation in response to sucrose might be suppressed in overexpressors of KIN10 (KIN10-ox) because SnRK1 activity is thought to inhibit growth and catabolism under conditions of starvation^12-14^, preventing seedlings from taking advantage of the additional sugars^1^. We reasoned that the converse might be true when SnRK1 activity is low, as occurs under sugar-replete conditions^10^. To investigate this, we measured the elongation of hypocotyls in response to sucrose in two T-DNA mutants of the KIN10 catalytic subunit of SnRK1 (GABI_579E09 or *akin10*^15^, and SALKseq_093965, a new allele named here *akin10*-2 for consistency) (Fig S1A). The full-length *KIN10* transcript is absent in these *akin10* and *akin10*-2 T-DNA lines (Fig. S1B). In the *akin10* mutant, there is a partial loss of phosphorylation of the SnRK1 target bZIP63, most likely due to reduced SnRK1 activity^15^. The remaining phosphorylation of bZIP63 in *akin10* is likely due to KIN11 activity^15^.

Supplementation of wild type seedlings with 3% (w/v) sucrose increased the hypocotyl length under short photoperiods but not under long photoperiods (Fig. 1A, B), as we reported previously^1^. Sucrose supplementation also increased the hypocotyl length of two *akin10* mutants under both short and long photoperiods (Fig. 1A, B). Under short photoperiods, sucrose caused a greater increase in hypocotyl length in *akin10* (6.51 mm longer, 224% increase) and *akin10*-2 (6.90 mm longer, 286% increase) compared with the wild type (3.75 mm longer, 67% increase) (Fig. 1A). This greater fold-change in hypocotyl length in the *akin10* mutants under these conditions is because the mutants had significantly shorter hypocotyls than the wild type in the absence of sucrose (Fig. 1A). Under long photoperiods, sucrose supplementation induced hypocotyl elongation in *akin10* mutants, which contrasted the wild type in which sucrose supplementation did not increase hypocotyl length (Fig. 1B). We found previously that sucrose supplementation can decrease the hypocotyl length of the Landsberg *erecta* background under long photoperiods^1^ but this did not occur in the Col-0 background used here (Fig. 1B), suggesting that there is some variation between accessions in this developmental response to sucrose.

Hypocotyls of *akin10* and *akin10*-2 mutants were significantly shorter than the wild type when cultivated in the absence of sucrose on 0.5MS meda (4 h photoperiods, *akin10* p < 0.001; *akin10*-2 p < 0.001; 16 h photoperiods, *akin10* p < 0.006; *akin10*-2 p < 0.001). In addition to changes in phytohormone signalling, the reduced hypocotyl elongation of *akin10* mutations might derive from altered seed quality^16^, attenuated seedling or embryo development as occurs in *tps1* knockouts^17^, altered circadian regulation^18^, or altered carbohydrate utilization^12, 19^.

The greater proportional increase in hypocotyl length that was caused by sucrose in *akin10* mutants compared with the wild type suggests that SnRK1 activity might contribute to suppression of hypocotyl elongation in response to sucrose. This is because KIN10 forms a catalytic subunit of SnRK1, and in the absence of this catalytic subunit there was an increase in the magnitude of sucrose-induced hypocotyl elongation. Although KIN10 and KIN11 are thought to confer kinase activity to the SnRK1 complex^12, 15^, *akin10* single mutants change the response of elongating hypocotyls to sucrose (Fig. 1). This indicates that KIN11 cannot completely replace KIN10 within the mechanisms underlying sucrose-induced hypocotyl elongation. This is consistent with the loss of SnRK1 kinase activity in the *akin10* single mutant^15^. An alternative interpretation is that there is some suppression of hypocotyl elongation in the *akin10* mutants in the absence of sucrose, and that this phenotype is lost in the presence of sucrose supplementation (Fig. 1A). Under long photoperiods, sucrose does not cause hypocotyl elongation in the wild type (Fig. 1B), which appears to be due to a combination of photoperiod and daily light input^1^. In comparison, there was sucrose-induced hypocotyl elongation in two *akin10* mutants under long photoperiods. However, under 16 h photoperiods sucrose induced a smaller increase in hypocotyl length in the *akin10* mutants than in *akin10* mutants under 4 h photoperiods. Therefore, as with the wild type^1^, photoperiod and/or daily light input influence the magnitude of sucrose-induced hypocotyl elongation in *akin10* mutants. This suggests that mechanisms additional to KIN10 activity within SnRK1 contribute to the photoperiod/daily light input within the response of elongating hypocotyls to sucrose. Such additional mechanisms could include the circadian oscillator, phototransduction pathways, and additional energy-sensing mechanisms.

**Figure legends**

**Figure 1.** Sucrose-induced hypocotyl elongation in wild type and *akin10* seedlings measured under (A) 4 h and (B) 16 h photoperiods. Seedlings were cultivated on control media (half strength Murashige and Skoog medium with 0.8% (w/v) agar; 0.5 MS), an equimolar osmotic control (sorbitol), or 3% (w/v) sucrose. Measurement of hypocotyl elongation was conducted as described by Simon et al. 2018. Statistical significance indicated for comparison between seedlings supplemented with 3% (w/v) sucrose and 87.6 mM sorbitol (osmotic control); analysis by univariate ANOVA followed by post-hoc Tukey analysis. Different letters indicate statistically-significant differences between means (*P* < 0.05); *n* = 20 +/- s.e.m.

**Figure S1.** Two T-DNA insertion mutants of *KIN10* decrease *KIN10* transcript abundance. (A) Position of two T-DNA insertions within KIN10 coding sequence. Sequence is within reverse orientation in genome and illustrated in forward orientation for clarity. (B) Comparison of *KIN10* transcript accumulation in Col-0 wild type, *akin10* and *akin10*-2 mutants. KIN10 transcript abundance measured using qRT-PCR, relative to *ELONGATION FACTOR 1 ALPHA (EF1ALPHA*, At5g60390) reference transcript^20^, n = 3 +/- s.e.m.

**Acknowledgements**

We thank BBSRC (UK) for funding (South-West Doctoral Training Partnership grant BB/J014400/1). We thank Prof. Alistair Hetherington for donating *akin10* mutants and Dr. Jean-Charles Isner for discussion about reference transcripts.

**Disclosure of potential conflicts of interest**

The authors declare no potential conflicts of interest

**References**

1. Simon NML, Kusakina J, Fernandez-Lopez A, Chembath A, Belbin FE, Dodd AN. The energy-signaling hub SnRK1 is important for sucrose-induced hypocotyl elongation. Plant Physiology 2018; 176:1299-310.

2. Kurata T, Yamamoto KT. *petit1*, a conditional growth mutant of Arabidopsis defective in sucrose-dependent elongation growth. Plant Physiology 1998; 118:793.

3. Takahashi F, Sato-Nara K, Kobayashi K, Suzuki M, Suzuki H. Sugar-induced adventitious roots in Arabidopsis seedlings. Journal of Plant Research 2003; 116:83-91.

4. Zhang Y, Liu Z, Wang L, Zheng S, Xie J, Bi Y. Sucrose-induced hypocotyl elongation of Arabidopsis seedlings in darkness depends on the presence of gibberellins. Journal of Plant Physiology 2010; 167:1130-6.

5. Liu Z, Zhang Y, Liu R, Hao H, Wang Z, Bi Y. Phytochrome interacting factors (PIFs) are essential regulators for sucrose-induced hypocotyl elongation in Arabidopsis. Journal of Plant Physiology 2011; 168:1771-9.

6. Stewart JL, Maloof JN, Nemhauser JL. PIF genes mediate the effect of sucrose on seedling growth dynamics. PLoS One 2011; 6:e19894.

7. Stewart Lilley JL, Gee CW, Sairanen I, Ljung K, Nemhauser JL. An endogenous carbon-sensing pathway triggers increased auxin flux and hypocotyl elongation. Plant Physiology 2012; 160:2261-70.

8. Zhang Z, Zhu J-Y, Roh J, Marchive C, Kim S-K, Meyer C, et al. TOR signaling promotes accumulation of BZR1 to balance growth with carbon availability in *Arabidopsis*. Current Biology 2016; 26:1854-60.

9. Zhang Y, Liu Z, Wang J, Chen Y, Bi Y, He J. Brassinosteroid is required for sugar promotion of hypocotyl elongation in Arabidopsis in darkness. Planta 2015; 242:881-93.

10. Zhang Y, Primavesi LF, Jhurreea D, Andralojc PJ, Mitchell RAC, Powers SJ, et al. Inhibition of SNF1-related protein kinase 1 activity and regulation of metabolic pathways by trehalose-6-phosphate. Plant Physiology 2009; 149:1860-71.

11. Yadav UP, Ivakov A, Feil R, Duan GY, Walther D, Giavalisco P, et al. The sucrose–trehalose 6-phosphate (Tre6P) nexus: specificity and mechanisms of sucrose signalling by Tre6P. Journal of Experimental Botany 2014; 65:1051-68.

12. Baena-González E, Rolland F, Thevelein JM, Sheen J. A central integrator of transcription networks in plant stress and energy signalling. Nature 2007; 448:938-42.

13. Baena-González E, Sheen J. Convergent energy and stress signaling. Trends in Plant Science 2008; 13:474-82.

14. Delatte TL, Sedijani P, Kondou Y, Matsui M, de Jong GJ, Somsen GW, et al. Growth arrest by trehalose-6-phosphate: An astonishing case of primary metabolite control over growth by way of the SnRK1 signaling pathway. Plant Physiology 2011; 157:160.

15. Mair A, Pedrotti L, Wurzinger B, Anrather D, Simeunovic A, Weiste C, et al. SnRK1-triggered switch of bZIP63 dimerization mediates the low-energy response in plants. eLife 2015; 4.

16. Radchuk R, Radchuk V, Weschke W, Borisjuk L, Weber H. Repressing the expression of the SUCROSE NONFERMENTING-1-RELATED PROTEIN KINASE gene in pea embryo causes pleiotropic defects of maturation similar to an abscisic acid-insensitive phenotype. Plant Physiology 2006; 140:263-78.

17. Gómez LD, Gilday A, Feil R, Lunn JE, Graham IA. AtTPS1-mediated trehalose 6-phosphate synthesis is essential for embryogenic and vegetative growth and responsiveness to ABA in germinating seeds and stomatal guard cells. The Plant Journal 2010; 64:1-13.

18. Shin J, Sánchez-Villarreal A, Davis AM, Du S-x, Berendzen KW, Koncz C, et al. The metabolic sensor AKIN10 modulates the Arabidopsis circadian clock in a light-dependent manner. Plant, Cell & Environment 2017; 40:997-1008.

19. Jossier M, Bouly J-P, Meimoun P, Arjmand A, Lessard P, Hawley S, et al. SnRK1 (SNF1-related kinase 1) has a central role in sugar and ABA signalling in Arabidopsis thaliana. The Plant Journal 2009; 59:316-28.

20. Nicot N, Hausman JF, Hoffmann L, Evers D. Housekeeping gene selection for real-time RT-PCR normalization in potato during biotic and abiotic stress. Journal of Experimental Botany 2005; 56:2907-14.

**Simon et al Supplemental Information**

**Figure S1.** Two T-DNA insertion mutants of *KIN10* decrease *KIN10* transcript abundance. (A) Position of two T-DNA insertions within KIN10 coding sequence. Sequence is within reverse orientation in genome and illustrated in forward orientation for clarity. (B) Comparison of *KIN10* transcript accumulation in Col-0 wild type, *akin10* and *akin10*-2 mutants. KIN10 transcript abundance measured using qRT-PCR, relative to *ELONGATION FACTOR 1 ALPHA (EF1ALPHA*, At5g60390) reference transcript^20^, n = 3 +/- s.e.m.
