# Supplementary figures and images for: Involvement of the SnRK1 subunit KIN10 in sucrose-induced hypocotyl elongation
Source: Plant Signal Behav. 2018 May 30;13(6):e1457913. doi: 10.1080/15592324.2018.1457913 (PMC6110359; doi:10.1080/15592324.2018.1457913)

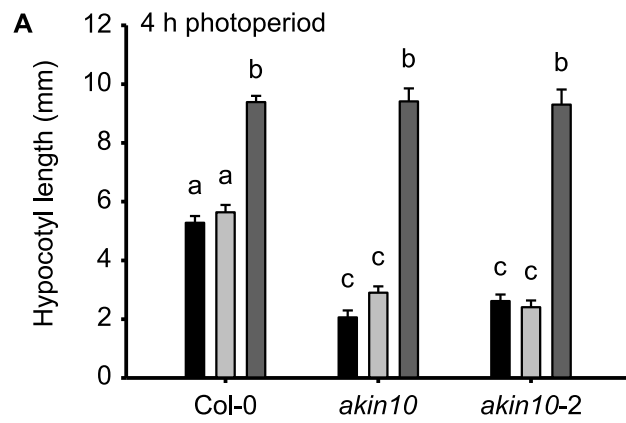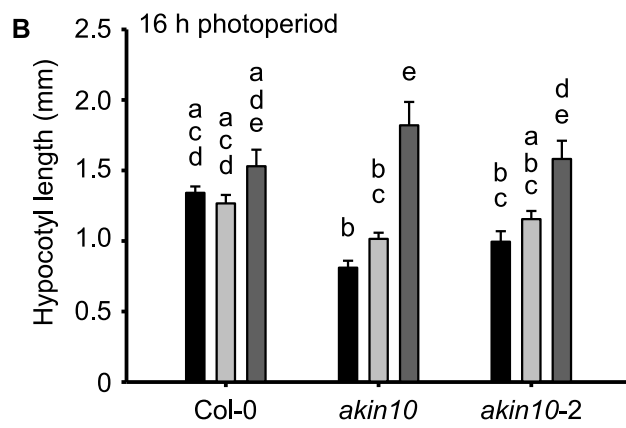

Control (0.5 MS media)  
87.6 mM sorbitol (osmotic control)  
3% (87.6 mM) sucrose

Supplement: Supplemental Material [file kpsb-13-06-1457913-s001.zip › Fig 1 v2.pdf]
